# Early High-dosage Atorvastatin Treatment Improved Serum Immune-inflammatory Markers and Functional Outcome in Acute Ischemic Strokes Classified as Large Artery Atherosclerotic Stroke: A Randomized Trial

**DOI:** 10.1097/MD.0000000000003186

**Published:** 2016-04-01

**Authors:** Antonino Tuttolomondo, Domenico Di Raimondo, Rosaria Pecoraro, Carlo Maida, Valentina Arnao, Vittoriano Della Corte, Irene Simonetta, Francesca Corpora, Danilo Di Bona, Rosario Maugeri, Domenico Gerardo Iacopino, Antonio Pinto

**Affiliations:** From the Internal Medicine and Cardioangiology Ward (AT, DDR, RP, CM, VDC, IS, FC, AP), Dipartimento Biomedico di Medicina Interna e Specialistica; Department of Experimental Medicine and Clinical Neurosciences (VA), Clinical Neurology ward; Department of Experimental Medicine and Clinical Neurosciences (RM, DGI), Neurosurgical Section, University of Palermo; and School and Chair of Allergology, Dipartimento delle Emergenze e Trapianti d’Organo (DDB), University of Bari, Bari Italy.

## Abstract

Statins have beneficial effects on cerebral circulation and brain parenchyma during ischemic stroke and reperfusion. The primary hypothesis of this randomized parallel trial was that treatment with 80 mg/day of atorvastatin administered early at admission after acute atherosclerotic ischemic stroke could reduce serum levels of markers of immune-inflammatory activation of the acute phase and that this immune-inflammatory modulation could have a possible effect on prognosis of ischemic stroke evaluated by some outcome indicators.

We enrolled 42 patients with acute ischemic stroke classified as large arteries atherosclerosis stroke (LAAS) randomly assigned in a randomized parallel trial to the following groups: Group A, 22 patients treated with atorvastatin 80 mg (once-daily) from admission day until discharge; Group B, 20 patients not treated with atorvastatin 80 mg until discharge, and after discharge, treatment with atorvastatin has been started.

At 72 hours and at 7 days after acute ischemic stroke, subjects of group A showed significantly lower plasma levels of tumor necrosis factor-α, interleukin (IL)-6, vascular cell adhesion molecule-1, whereas no significant difference with regard to plasma levels of IL-10, E-Selectin, and P-Selectin was observed between the 2 groups.

At 72 hours and 7 days after admission, stroke patients treated with atorvastatin 80 mg in comparison with stroke subjects not treated with atorvastatin showed a significantly lower mean National Institutes of Health Stroke Scale and modified Rankin scores.

Our findings provide the first evidence that atorvastatin acutely administered immediately after an atherosclerotic ischemic stroke exerts a lowering effect on immune-inflammatory activation of the acute phase of stroke and that its early use is associated to a better functional and prognostic profile.

## INTRODUCTION

### Background and Objectives

In addition to their lipid-lowering effects, it has been speculated that statins may also have beneficial effects on cerebral circulation and brain parenchyma during ischemic stroke and reperfusion. Asahi et al^1^ reported that statins increased endothelial nitric oxide synthase (eNOS) and tissue plasminogen activator (tPA) mRNA levels, but did not change mRNA levels of plasminogen activator inhibitor-1 (PAI-1) and that in eNOS knockout mice, atorvastatin reduced the volume of ischemic tissue and improved neurologic outcomes after arterial occlusion by blood clot emboli. Aslanyan et al^2^ reported that statin use was associated with reduced mortality at 1 month during the follow-up after stroke, whereas in patients with recent stroke or Transient ischemic attack and without known coronary heart disease, 80 mg/day of atorvastatin reduced the overall incidence of strokes and of cardiovascular events, despite a small increase in the incidence of hemorrhagic stroke period.^3^

Furthermore, Pignatelli et al^4^ recently showed the first evidence that atorvastatin acutely and simultaneously decreases oxidative stress and platelet activation by directly inhibiting platelet Nox2 and ultimately platelet isoprostanes and thromboxane A2, providing a rationale for the use of statins to prevent or modulate thrombosis.

Inflammation plays a crucial role in mediating all the stages of the atherosclerosis process.^6^ Similarly, thrombosis and defective fibrinolysis may also contribute to the progression of atherosclerotic lesions.^5^ Interestingly, both mechanisms might have a relevant role in the pathogenesis of intracranial large artery atherosclerosis and ischemic stroke, particularly in atherosclerotic subtype.

Our group showed that patients with cardioembolic and atherothrombotic stroke subtypes showed significantly higher median plasma levels of tumor necrosis factor (TNF)-α, interleukin (IL-6), IL-1β, whereas the lacunar subtype showed significantly lower median plasma levels of TNF-α, IL-6, and IL-1β^5,7,8,9^ and that immune-inflammatory marker plasma levels are significantly related to ischemic lesion volume.^9^

Whereas recent data suggest that inflammatory reactions are involved in the pathogenesis and progression of cerebral ischaemia,^10^ no study has evaluated effects of atorvastatin 80 mg/day after a recent stroke on outcome and on immune-inflammatory markers to evaluate acute antithrombotic, anti-inflammatory, and neuroprotective effects of atorvastatin in acute cerebrovascular event setting.

Thus, we hypothesized that acute administration of high-dose atorvastatin early after an acute ischemic stroke could be beneficial because of its reported anti-inflammatory, antioxidant, and antithrombotic properties.

The primary objective of this study was to evaluate the effects of atorvastatin 80 mg/day in vivo on immune-inflammatory markers and on functional outcome in patients with recent acute ischemic stroke classified as atherothrombotic not treated with thrombolytic treatment.

## MATERIALS AND METHODS

The trial was carried out in 2 parts. Part 1 assessed differences in serum levels of some immune-inflammatory markers as a measure of the anti-inflammatory activity of early treatment with atorvastatin; Part 2, used 2 outcome measures representing different aspects of recovery from stroke to assess whether early treatment with atorvastatin resulted in a better clinical benefit at 72 hours and 7 days (differences in National Institutes of Health Stroke Scale [NIHSS] score) and at 7 days (Rankin score).

## HYPOTHESES AND DESIGN

Part 1 was designed to test whether atorvastatin had effects on lowering serum levels of some immune-inflammatory markers at 72 hours and 7 days after stroke in patients treated with atorvastatin as compared with those not treated with atorvastatin.

Part 2 was designed to test whether atorvastatin treatment is associated to a lower mean NIHSS score at 72 hours and 7 days after stroke and a lower mean Rankin score at 7 days after stroke in a greater proportion of patients treated with atorvastatin as compared with those not treated with atorvastatin.

## TRIAL DESIGN

### Interventions: Treatment Protocol

This randomized trial has a parallel design.

### Eligibility Criteria

To be eligible for the study, patients had to have an ischemic stroke with a clearly defined time of onset, a deficit measurable on the NIHSS, and a baseline computed tomographic (CT) scan of the brain that showed no evidence of intracranial hemorrhage and classified as Large Atherosclerotic Stroke (LAAS) according TOAST classification and that have not been treated with thrombolysis.

We excluded patients treated with trombolysis owing to the fact that trombolysis treatment is the unique effective treatment in acute stroke setting with well-reported rapid effects on outcome indicators and immunoinflammatory markers that could represent a possible bias in evaluation of effects of atorvastatin treatment in early post acute phase after an acute ischemic stroke.

Patients with inflammatory or infectious diseases, cancer, hematological diseases, and severe renal or liver failure, as well as those who were under treatment with anti-inflammatory drugs, were excluded.

Stroke was defined by focal neurological signs or symptoms thought to be of vascular origin that persisted for N24 h, confirmed by brain CT and/or MRI in baseline conditions.^11^

### Randomization

Patients were randomly assigned by the use of sequentially numbered boxes (prepared before starting the study by a computerized, non-alternating sequence) to the following groups: Group A, treatment with atorvastatin 80 mg (once-daily) from admission day and maintained after discharge as indicated by SPARCL trial^3^; Group B, no treatment with atorvastatin 80 mg until discharge, after discharge, treatment with atorvastatin has been started as indicated by SPARCL trial.^3^

In patients with disorder of consciousness or in patients with swallowing deficit, atorvastatin has been administered through a nasogastric tube after solubilitation of tablets.

We evaluated plasma levels of IL-1β, TNF-α, IL-6 and IL-10, E-selectin, P-selectin, soluble intercellular cell adhesion molecule-1 (sICAM-1), and soluble vascular cell adhesion molecule (sVCAM-1) as markers of immune-inflammatory activation, von Willebrand factor (VWF) plasma levels as a marker of endothelial dysfunction, and TPA-antigen and PAI-1 plasma levels as thrombotic/fibrinolytic markers.

These laboratory evaluations have been done at 72 h and at 7 days after symptom onset.

### Outcome Measures

Two outcome measures were selected on the basis of their reliability: neurological deficit score on admission and at 72 hours and 7 days after stroke was evaluated by the NIHSS.^12^ The NIHSS is a tool used by healthcare providers to objectively quantify the impairment caused by a stroke. The NIHSS is composed of 11 items, each of which scores a specific ability between a 0 and 4. For each item, a score of 0 typically indicates normal function in that specific ability, whereas a higher score is indicative of some level of impairment. The individual scores from each item are summed to calculate a patient's total NIHSS score. The maximum possible score is 42, with the minimum of 0.

Disability at 7 days was evaluated by modified Rankin Scale (mRS)^13^ that is a commonly used scale for measuring the degree of disability or dependence in the daily activities of people who have suffered a stroke.

The scale runs from 0 to 6, running from perfect health without symptoms to death:

1. No symptoms.
2. No significant disability: able to carry out all usual activities, despite some symptoms.
3. Slight disability: able to look after own affairs without assistance, but unable to carry out all previous activities.
4. Moderate disability: requires some help, but able to walk unassisted.
5. Moderately severe disability: unable to attend own bodily needs without assistance, and unable to walk unassisted.
6. Severe disability: requires constant nursing care and attention, bedridden, incontinent.
7. Dead.

### Study Settings

The patient and control data were collected at Internal Medicine and Cardioangiology Ward, Policlinico P. Giaccone Hospital of Palermo.

We enrolled patients with a diagnosis of acute ischemic stroke admitted to the Internal Medicine Department at the University of Palermo between December 2013 and may 2015 (date recruitment range: December 1, 2013–May 30, 2015).

All recruited patients have been evaluated after recruitment for outcome evaluation at 72 hours and 7 days after stroke onset between December 2013 and June 2015 (date follow up evaluation range: May 9, 2013–June 14, 2014).

### Risk Factor Evaluation

Cardiovascular risk factors were evaluated for both subjects and controls according the following criteria: type 2 diabetes mellitus was determined using a clinical-based algorithm that considered age at onset, presenting weight and symptoms, family history, onset of insulin treatment, and history of ketoacidosis.

Atrial fibrillation was diagnosed when present on the admission ECG.

Hypertension was present when a patient had received antihypertensive treatment before admission or when hypertension was diagnosed during the hospital stay by repeated detection of blood pressure ≥160/95 mmHg. Hypercholesterolemia was defined as total serum cholesterol ≥200 mg/dL or on the basis of a clinical history of hypercholesterolemia or statin treatment.

### Definition of Stroke and Stroke Subtypes

The type of acute ischemic stroke was classified according to the TOAST classification^14^: LAAS; CardioEmbolic Infarct; LACunar infarct; stroke of Other Determined Etiology; stroke of UnDetermined Etiology.

#### LAAS Patients Selection

These patients will have clinical and brain imaging findings of either significant (≥50%) stenosis or occlusion of a major brain artery or branch cortical artery, owing to atherosclerosis. Clinical findings include those of cerebral cortical impairment (aphasia, neglect, restricted motor involvement, etc) or brain stem or cerebellar dysfunction. Cortical or cerebellar lesions and brain stem or subcortical hemispheric infarcts >1.5 cm in diameter on CT or MRI are considered to be of potential large artery atherosclerotic origin. Supportive evidence by duplex imaging or arteriography of a stenosis of >50% of an appropriate intracranial or extracranial artery is needed. Diagnostic studies should exclude potential sources of cardiogenic embolism.^2^

### Patient Evaluation and Blood Sample Collection

All the ischemic stroke patients underwent: medical history with recording of potential stroke risk factors, blood and coagulation tests, 12-lead ECG, 24-h electrocardiography monitoring, transthoracic echocardiography, carotid ultrasound, brain CT or MRI at admission (repeated between the third and the seventh day of stroke onset).

Blood samples were obtained in the nonfasting state. After 10 minutes of rest in the supine position, vital signs were recorded and blood samples were collected from the antecubital vein. Ethylediaminetetraacetic acid-anticoagulated peripheral blood was drawn from each patient within 72 hours (accepted range 72 ± 3 hours) and at 7 days from symptom onset after sample collection serum and plasma were immediately separated by centrifugation and stored in aliquots at –80°C until analysis.

We evaluated plasma levels of IL-1β, TNF-α, IL-6 and IL-10, E-selectin, P-selectin, sICAM-1, and sVCAM-1 as markers of immunoinflammatory activation, VWF plasma levels as a marker of endothelial dysfunction, TPA-antigen and PAI-1 plasma levels as thrombotic/fibrinolytic markers.

These laboratory evaluations have been done at 72 h and at 1 week after symptom onset.

IL-1β, TNF-α, IL-6 and IL-10, and VWF antigen were measured using a sandwich ELISA (Human IL-1β, TNF-α, IL-6 and IL-10 Quantikine, R&D Systems, VWF ELISA kitdurian, Instrumentation Laboratory, Milano, Italy); VCAM-1, ICAM-1, E-selectin, P-selectin, PAI-1 and TPA antigen were measured by commercial bioimmunoassay (Human sICAM-1, sVCAM-I, sE-selectin and sP-selectin Parameter, Quantikine, R&D Systems, Gentaur AssayMax Human Plasminogen Activator Inhibitor-1 [PAI-1] ELISA Kit, Gentaur AssayMax Tissue Plasminogen Activator [TPA] ELISA Kit).

The minimum detectable concentrations for the diagnostic tests were: TNF alpha, 1.6 pg/mL; IL-1β, b1 pg/mL; IL-6:b0. 0 pg/mL; IL-10, 3.9 pg/mL; ICAM-1, 0.35 ng/mL;VCAM-1, 0.6 ng/mL; E-Selectin, 0.1 ng/mL; P-Selectin, 0.5 ng/mL; vWF, 1.0%; TPA, 0.3 pg/mL; PAI-1, b50 pg/mL. Intra-assay and inter-assay coefficients of variation were: TNF alpha 4.2% and 4.6%; IL-1β 3.3% and 4.2%; IL-6 1.6% and 3.3%; IL-10 4.3% and 7.5%; ICAM-1 4.8% and 6.1%; VCAM-1 3.5% and 7.7%; E-Selectin 4.8% and 5.7%; P-Selectin 4.9% and 8.8%; vWF 5% and 10%; TPA 4.8% and 5%; PAI-1 5.7% and 8.3%.

### Primary Outcome

Primary outcome is the differences in mean immuno-inflammatory marker serum levels between group A and B at 72 hours and 7 days after stroke.

### Secondary Outcomes

Secondary outcomes are as follows:

1. Differences in mean NIHSS at 72 hours after stroke between group A and B.
2. Differences in mean NIHSS at 7 days after stroke between group A and B.
3. Difference in mean Rankin score at 7 days after stroke between group A and B.

For trial design and manuscript preparation we followed CONSORT reporting guidelines.

### Sample Size and Statistical Analysis

For power calculations, we assumed a decrease of 30% of chosen serum cytokines (IL-6, TNF-α, IL-1β) if at least 20 patients were assigned to each group; this would give a power of >80% to detect differences of these magnitudes among groups^15^ and to detect prognostic differences among the 2 groups of enrolled subjects because of changes of immunoinflammatory markers and of outcome indicators.

Categorical variables are reported as counts (percentage) and continuous variables as mean andSD unless otherwise indicated. Differences between percentages were assessed by *χ*^2^ test or Fisher exact test. A Student unpaired *t* test and Pearson correlation analysis were used for normally distributed continuous variables. Appropriate nonparametric tests (Mann-Whitney *U* test or Spearman rank correlation test) were used for all the other variables. Interventional study data were analyzed for the assessment of treatment effect on immunoinflammatory variables plasma levels and outcome indicators (NIHSS and mRS) and 1 within-subject factor (time at 2 levels: after 72 hours and 7 days after the beginning of the treatment). As covariates, we considered the possible random differences in age, sex, body mass index, blood pressure, and blood lipid profile between the 2 groups. Values of *P* < 0.05 were regarded as statistically significant. All calculations were made with Statistical 7 software for Windows (StatSoft, Tulsa, OK). Statistical informations of our manuscript have been evaluated by an expert.

## RESULTS

Among 113 patients admitted to our ward with a diagnosis of acute ischemic stroke in the enrolment period, we enrolled 99 patients (53 males and 39 females) with acute ischemic stroke.

Fourteen patients were excluded for the presence of exclusion criteria (5 for previous inflammatory or infectious diseases, 4 for cancer, 2 for hematological diseases 3 for undertreatment with anti-inflammatory drugs).

Among stroke patients were randomized to treatment protocol only subjects classified as LAAS a TOAST Classification. Forty-two patients were classified as LAAS.

### Randomization

**FIGURE 1:**
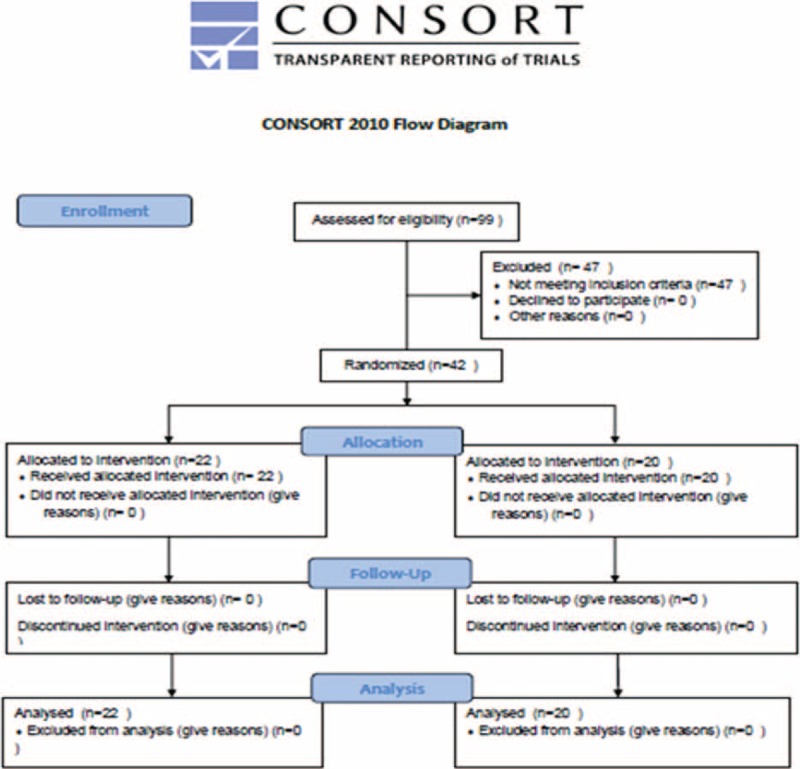
Consort flow diagram.

Among subjects classified as LAAS, 22 patients (Group A) were randomized to treatment with atorvastatin 80 mg/day and 20 patients (Group B) were randomized to no treatment with atorvastatin in the acute phase of stroke **(**see Figure 1: flow diagram of participants).

No significant difference was observed between the 2 groups with regard to cardiovascular risk factor prevalence (hypertension, diabetes, dyslipidemia, previous stroke), WBC and platelet count, serum lipid profile, and with regard to cardiovascular therapy before stroke (antiplatelets, antihypertensive drugs, statins).

No patient of both group interrupted in drug administration because of muscular or hepatic toxicity or other collateral effects. Among stroke patients treated with atorvastatin 80 mg, no significant differences have been observed either at 72 hours or 7 days after admissions with regard to serum levels of alanine transaminase, aspartate transaminase, gamma-glutamyltransferase, and alkaline phosphatase.

### Characteristics of Enrolled Patients

Demographic, laboratory, and clinical variables of stroke patients with LAAS TOAST subtype are listed in Table 1.

**TABLE 1:**
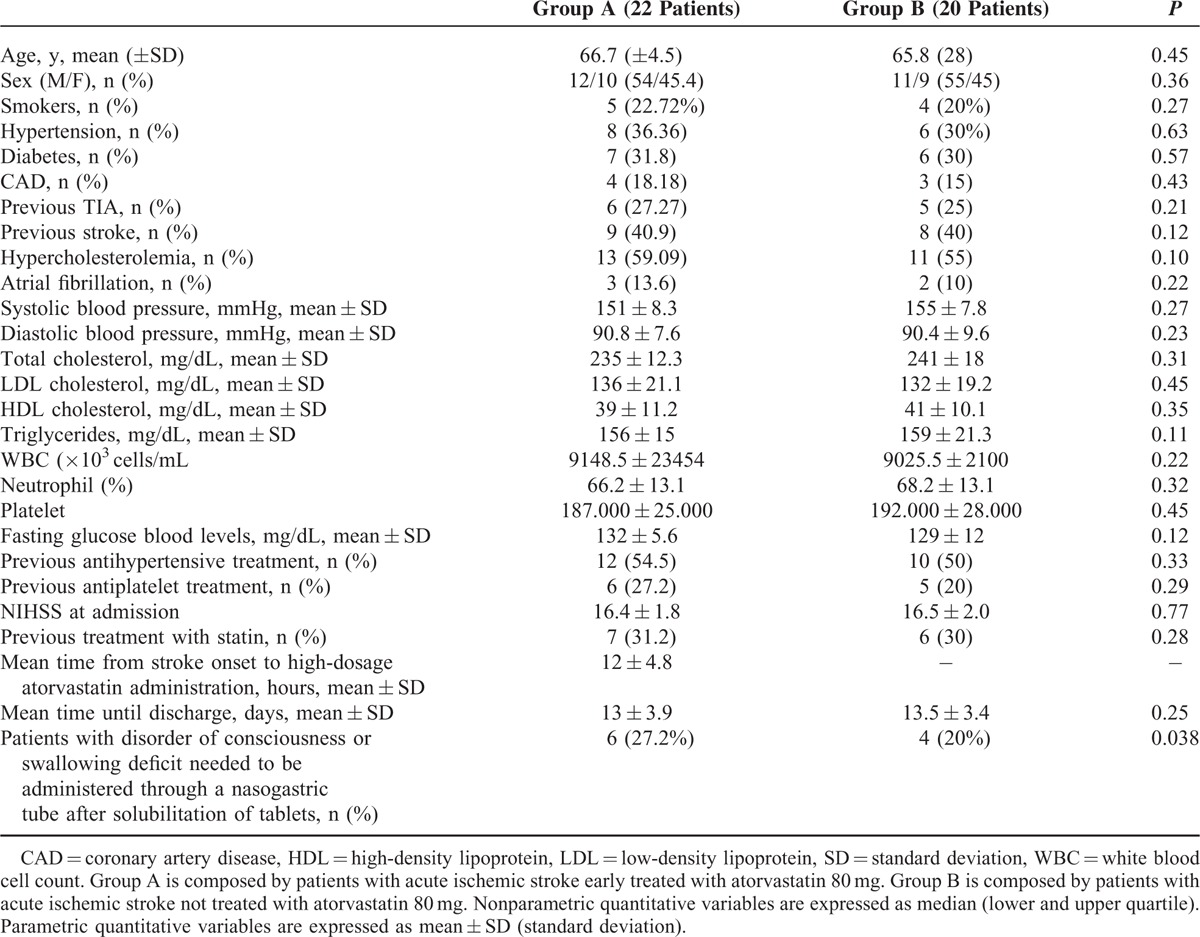
Baseline Characteristics of Patients With Acute Ischemic Stroke Treated With Atorvastatin (Group A) and Not Treated With Atorvastatin (Group B)

Mean time from stroke onset to statins administration was 12 ± 4.8 hours in group A.

No significant difference with regard to low-density lipoprotein cholesterol (136 ± 21.1 vs 134 ± 1.8, *P* = 0.23) and high-density lipoprotein cholesterol (39 ± 11.2 vs 40.02 ± 1.1; *P* = 0.32) values was observed in subjects of group A after 7 days of atorvastatin treatment.

### Inflammatory Marker Changes at 72 hours After Stroke

**FIGURE 2:**
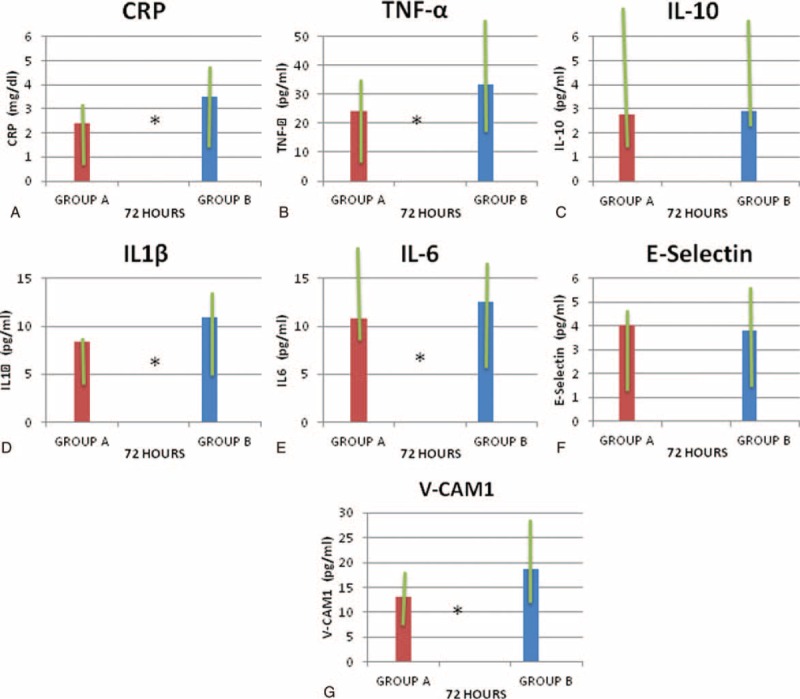
Median (lower and upper quartile) of serum levels of C-reactive protein (CRP) (A); tumor necrosis factor (TNF-α) (B); interleukin-10 (IL-10) (C); IL-1β (D); IL-6 (E); E-selectin (F); and vascular cell adhesion molecule-1 (VCAM-1) (**G**) at 72 hours after acute event in subjects with acute ischemic stroke early treated with atorvastatin 80 mg (Group A) and not treated with atorvastatin 80 mg (Group B). ^∗^*P* *<* *0.05.*

With regard to inflammatory marker plasma levels at 72 hours after acute ischemic stroke, subjects of group A showed in comparison with subjects of group B significantly lower plasma levels of CRP (2.4 [0.8–2.9] mg/dL vs 3.5 [1.2–4.6] mg/dL, *P* = 0.035; TNF-α (24.3 [7.75–38] pg/mL vs 33.5 (10.25–44) pg/mL; *P* = 0.023), IL-1β (8.4 [4–9.8] pg/mL vs 11 [5–14] pg/mL, *P* = 0.038), IL-6 (10.8 [7.2–19) pg/mL vs 12.6 [8.5–18] pg/mL, *P* = 0.010), VCAM-1 (19 [12.5–27] pg/mL vs 23 [14.6–36] pg/mL, *P* = 0.025), and ICAM-1 (13 [7.7–19] pg/mL vs 18.7 [10.2–29] pg/mL, *P* < 0.0001) (see Table 2 and Figure 2 A–G).

**TABLE 2:**
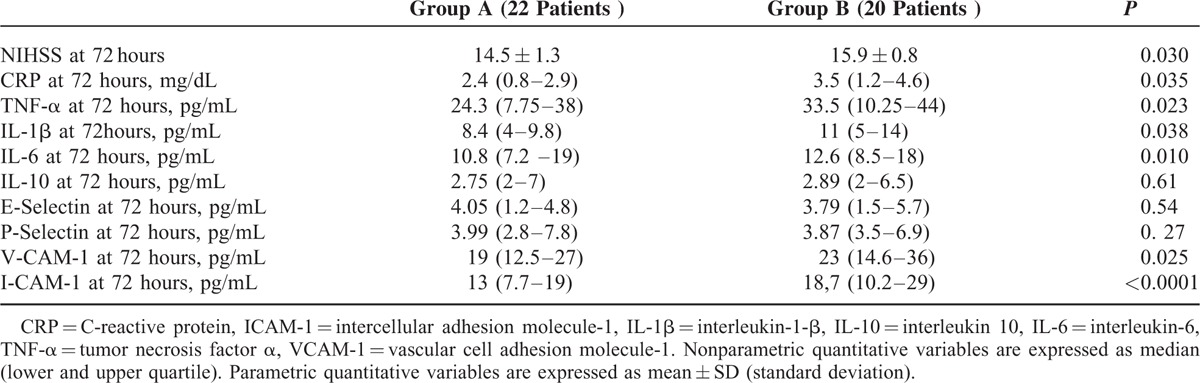
Outcome Indicators and Immunoinflammatory Markers at 72 Hours After Acute Event in Subjects With Acute Ischemic Stroke Early Treated With Atorvastatin 80 mg and Not Treated With Atorvastatin 80 mg

### Inflammatory Marker Changes at 7 Days After Stroke

**FIGURE 3:**
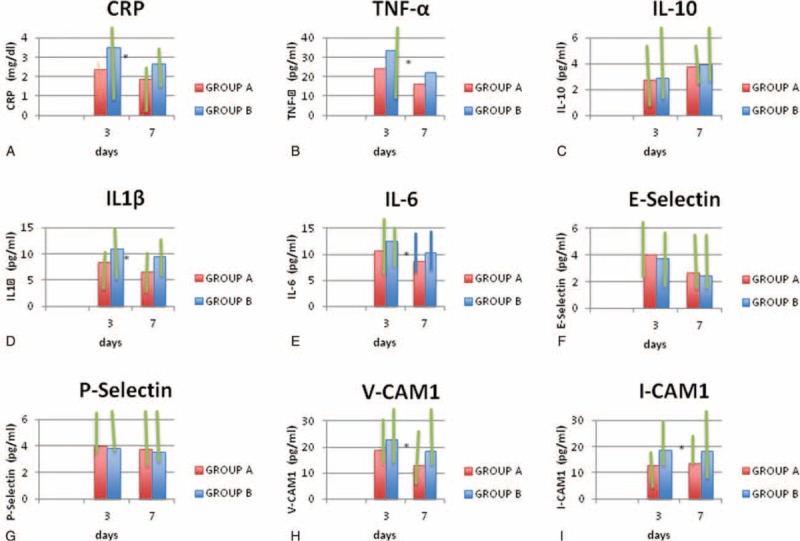
Serum median (lower and upper quartile) levels of C-reactive protein (CRP) (A); tumor necrosis factor (TNF-α) (B); interleukin-10 (IL-10) (C), IL-1β (D); IL-6 (E); E-selectin (F); P-selectin (G); vascular cell adhesion molecule-1 (VCAM-1) (H); and intercellular adhesion molecule-1 (ICAM-1) (I) at 72 hours and 7 days after acute event in subjects with acute ischemic stroke early treated with atorvastatin 80 mg (Group A) and not treated with atorvastatin 80 mg (Group B). ^∗^*P* *<* *0.05.*

With regard to inflammatory marker plasma levels at 7 days after acute ischemic stroke, subjects of group A in comparison with subjects of group B showed significantly lower plasma levels of CRP (1.9 [0.7–2.0] mg/dL vs 2.7 [1.5–3.3] mg/dL, *P* = 0.043), TNF-α (16.3 [6.75–26] pg/mL vs 22.3 [8.75–38] pg/mL, *P* = 0.031), IL-1β (6.6 [3–11] pg/mL vs 9.5 [6.8–12] pg/mL, *P* = 0.022), IL-6 (8.7 [5.5–14] pg/mL vs 10.4 [6.7–17] pg/mL, *P* = 0.023), VCAM-1 (13 [7.7–29] pg/mL vs 18.7 [7.8–34] pg/mL, *P* = 0.025), ICAM-1 (13.5 [11.5–26] pg/mL vs 18.5 [16.5–31] pg/mL *P* = 0.039) (see Table 3 and Figure 3 A–H).

**TABLE 3:**
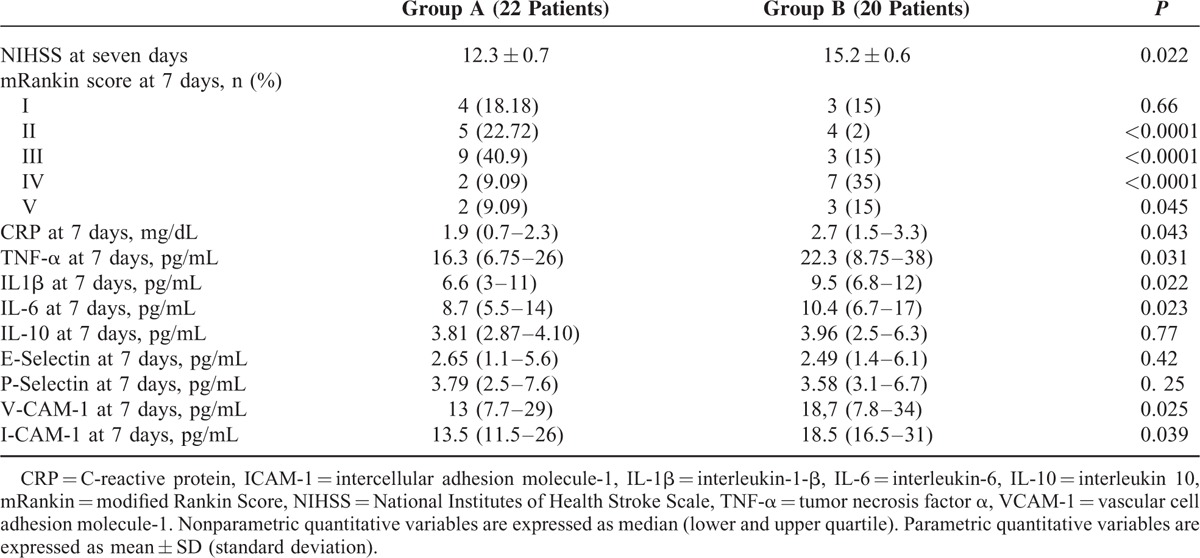
Outcome Indicators and Immunoinflammatory Markers at 7 Days After Acute Event in Subjects With Acute Ischemic Stroke Early Treated With Atorvastatin 80 mg and Not Treated With Atorvastatin 80 mg

No significant difference with regard to plasma levels of IL-10, E-selectin, and P-selectin was observed between subjects treated with atorvastatin and those not treated with atorvastatin both at 72 h and at 7 days after acute ischemic stroke (see Tables 2 and 3).

### Outcome Indicators Change at 72 Hour After Stroke

With regard to chosen outcome indicators, stroke patients treated with atorvastatin 80 mg at 72 hours after admission showed a significantly lower mean NIHSS score in comparison with stroke subjects not treated with atorvastatin (14.5 ± 1.3 vs 15.9 ± 0.8; *P* = 0.030); analogously at 7 days after admission, stroke patients treated with atorvastatin showed a significantly lower mean NIHSS score 80 mg in comparison with stroke subjects not treated with atorvastatin (12.3 ± 0.7 vs 15.2 ± 0.6; *P* = 0.022) (see Table 3).

### Outcome Indicators Change at 7 Days After Stroke

With regard to mRS, at 7 days after admission, stroke patients treated with atorvastatin 80 mg in comparison with stroke subjects not treated with atorvastatin showed a higher percentage of patients were classified as II class (22.72% vs 2%; *P* < 0.0001) and as III class (40.9% vs 15%; *P* < 0.0001), whereas a lower percentage of patients were classified as IV class (9.09% vs 35%; *P* < 0.0001) and V (9.09% vs 15 %; *P* = 0.045) (see Table 3).

Patients with highest reduction of immune-inflammatory markers at 7 days are also patients with a better change of outcome indicators at 7 days. Stroke subjects who at 7 days after admission showed mean serum levels of TNF-α >20 pg/mL showed a mean NIHSS of 17.5 ± 1.0, those with mean serum levels of IL-1β >7 pg/mL showed a mean NIHSS of 17.8 ± 1.8, and those with mean serum levels of Il-6 >9 pg/mL showed a mean NIHSS of 18.0 ± 1.4, whereas patients who at 7 days after admission showed mean serum levels of TNF-α <20 pg/mL showed a mean NIHSS of 12.8 ± 1.7, those with mean serum levels of IL-1β <7 pg/mL showed a mean NIHSS of 10.1 ± 1.5 and those with mean serum levels of IL-6 <9 pg/mL showed a mean NIHSS of 11.3 ± 1.6. Furthermore, stroke subjects who at 7 days after admission showed mean serum levels of TNF-α >20 pg/mL showed a mean Rankin score of 3.8 ± 0.9, those with mean serum levels of IL-1β >7 pg/mL showed a mean Rankin score of 4 ± 0.5, and those with mean serum levels of Il-6 >9 pg/mL showed a mean Rankin score of 3.9 ± 0.4, whereas patients who at 7 days after admission showed mean serum levels of TNF-α <20 pg/mL showed a mean Rankin score of 2 ± 0.8, those with mean serum levels of IL-1β <7 pg/mL showed a mean Rankin score of 2.9 ± 0.3, and those with mean serum levels of IL-6 <9 pg/mL showed a mean Ramkin score of 2.8 ± 0.3.

## DISCUSSION

Our findings provide the first evidence that atorvastatin acutely administered early after an atherosclerotic ischemic stroke exerts a lowering effect on serum markers immune-inflammatory activation of the acute phase of stroke and that its use is associated to a better outcome profile in terms of acute neurological deficit and disability grade.

We cannot affirm that cytokine serum level reduction in our enrolled patients with acute ischemic stroke is only because of atorvastin treatment owing to the fact that there exists a progressive lowering of cytokines after brain ischemic events. Thus, our interpretation of this finding is related to a possible overlapping anti-inflammatory efficacy of atorvastatin to the normal dynamics of inflammatory markers after acute ischemic stroke. Nevertheless, the aim of our study was not to show a reduction of serum levels of cytokines compared with baseline levels, but to evaluate differences in serum levels of cytokines at 72 hours after stroke and a more significant reduction of these levels at 7 days after stroke and their relationship with outcome differences in subjects treated with early oral atorvastatin and in subjects not treated with atorvastatin.

No significant changes in lipid blood profile were observed at 7 days after stroke onset; thus, our findings appear independent from blood lipid changes, perhaps owing to the fact that 7 days of treatment is a too short observation period to detect significant change in lipid blood profile.

Experimental and clinical studies demonstrated that statins exert an antioxidant effect with a mechanism potentially involving downregulation of Nox2 and upregulation of nitric oxide synthase coupling.^16,17^

Previous studies using several markers of platelet activation in patients with stable or unstable cardiovascular disease have already shown that statins exert an antiplatelet effect that was early and apparently independent from the cholesterol-lowering property of statins.^18,19,20^

Moreover, recently, Pignatelli et al^4^ provided the first evidence that atorvastatin acutely and simultaneously decreases oxidative stress and platelet activation by directly inhibiting platelet Nox2 and ultimately platelet isoprostanes and thromboxane A2, whereas clinical^21–30^ and experimental^22–26^ evidence suggest that part of the beneficial effects of statins are independent of their lipid-lowering properties, their so-called pleiotropic effects.^31^

A recent study^32^ showed that in 129 patients receiving statins, 123 initiated statins within 4 weeks, and in 600 patients not on statins, multivariate logistic regression analysis demonstrated that poststroke statins were associated with a significant probability of a favorable outcome at 12 weeks and mRS, whereas prestroke statins demonstrated a trend toward significance.

These findings could possibly explain our results showing that high-dose atorvastatin administered early after an atherosclerotic stroke is significantly associated with a lower degree of serum immune-inflammatory markers both at 72 hours and 7 days after stroke and that this lower immune-inflammatory degree is possibly related to a better prognosis evaluated by means outcome indicators such as NIHSS and mRS scores.

Several explanations could be considered to explain our findings concerning significant effects of atorvastatin on immunoinflammatory activation of the acute phase and on functional outcome indicators at 72 hours and 7 days after stroke onset.

In addition to their activities as cholesterol-lowering drugs, statins are known to have complex pleiotropic effects, including anti-inflammatory, immunomodulatory, and endothelial function-enhancing effects both in vitro and in human studies.^33^ Statins inhibit the mevalonate pathway, which involves the synthesis of isoprenoids and the intracellular trafficking of membrane-associated proteins such as G-proteins. GTP-binding proteins have crucial roles in intracellular inflammatory signaling by acting as molecular switches for various protein kinases. Specific targets, including Ras, Rho, and Rac subfamilies, are thought to be important in systemic inflammatory syndromes, such as sepsis, because of their key roles in intracellular signaling.^34–35^

Furthermore, a very recent study reported how therapy with statins, either previous or early initiation, after an ischemic stroke, could improve the survival and readmission rates by lowering both cholesterol and high-sensitivity C-reactive protein levels.^36^

In vitro and animal studies have shown that statins reduce the release of TNF-α and IL-1b.^35,37^ In a murine model of cerebral malaria,^37^ treatment with lovastatin intravital examination of pial vessels of infected animals demonstrated a reverse of rolling and adhesion of leukocytes to inflamed endothelium and reduction of ICAM-1 and CD11b mRNA levels and a reduction of IL-1b, TNF-a, and IL-12 levels that were elevated in the brains of mice on post infection period.

Statins could also impair immunoinflammatory cell trafficking. Lovastatin changed the expression of specific cytokines, promoting a shift from a Th1 to a Th2 response.^38–42^ Thus, statins would thus modulate the immune system through coordinated activity in various arms of the immune reaction, including terminal differentiation of immune cells, as well as the regulation of the molecules that regulate these cells to the sites at which they exert their immunomodulatory activity.

Furthermore, statins in brain may be both neuroprotective and regenerative, and could lead to a new direction in understanding the potential therapeutic efficacy of statins in AD and in particular atorvastatin treatment has been reported as neuroprotective against cell degeneration induced by amyloid-beta,^43^ reducing inflammatory and oxidative responses and increasing the expression of glutamatergic transporters.^44,45^

Recent experimental data from a murine model of ischemic stroke demonstrate that prophylactic statin therapy augments cerebral blood flow, reduces infarct size by 30%, and improves neurological outcome in normocholesterolemic animals.^43^ Statins have been shown to reduce infarct size in experimental animal models of stroke by means a summa of neuroprotective action such as upergulation of endothelia _e_NOS and inhibition of inducible inducible nitric oxide synthase (_i_NOS), lowering neuronal inflammation and amelioration of ischemic oxidative stress in brain.

Furthermore, statins may also exert antithrombotic activities mediated by changes of the coagulation system.^44,45,46^

A previous pilot, double-blind, randomized, multicenter clinical trial^47^ has been conducted to study for the first time safety and efficacy of simvastatin in the acute phase of ischemic stroke in terms of the evolution of several inflammation markers (IL-6, IL-8, IL-10, monocyte chemoattractant protein-1, ICAM-1, VCAM-1, C-reactive protein, TNF-α, E-selectin, L-selectin, and nitrites + nitrates) and neurological outcome was evaluated at baseline, day 1, 3, 5, 7, and 90. Authors used simvastatin at an initial dose of 40 mg/day for the first week followed by a dose of 20 mg/day until day 90. No differences were found among the biomarkers studied regarding treatment allocation, although simvastatin patients improved significantly by the third day. These findings contrast with our results probably owing to differences in statin dosage used in the trial and owing to the fact that we used high dosage of atorvastatin, a statin with well-proven anti-inflammatory and neuroprotective antinflammatory properties.

### Limitations

A limitation of our study is the small sample size suggesting us a certain caution when interpreting our results.

Among possible limitations of our study, we acknowledge the potential weakness of the open design of the study; however, randomization and blind analysis of laboratory variables were likely to limit this bias.

Another possible limitation is offered by high atorvastatin dosage that we used, but we chose to use high dosage of atorvastatin owing to the fact that other experimental studies^4^ that evaluated the role of atorvastatin as antioxidant and antithrombotic agents and clinical studies that reported the beneficial effects of atorvastatin in secondary prevention, such as Stroke Prevention by Aggressive Reduction in Cholesterol Levels (SPARCL) study^3^ used high dosage (80 mg) of atorvastatin, thus making plausible to test only this high dosage in our study.

## CONCLUSIONS

### Interpretation and Generalizability

In conclusion, our findings provide a rationale for generalizability of the early use of high-dosage atorvastatin in patients with acute ischemic stroke as a possible neuroprotective treatment. Further study is necessary to see whether a similar effect can be achieved and maintained in a longer follow-up examination without toxicity effects that we did not observe at a short observation period. A Possible external validity or generalizability to other circumstances could be related to the possibility of evaluation of a possible neuroprotective role of statins such as atorvastatin in other circumstances of acute neurological disorders such as thraumatic brain insult or cerebral hemorrhages, but further study is needed to evaluate atorvastatin role in this clinical setting. Furthermore, although the biological plausibility of our findings, since the sample size was assessed for primary end point our conclusion regarding the associated improvement of clinical outcome should be only considered as an interest hypothesis-generating result that should encourage the development of a further larger, multicenter, and controlled randomized trials.
